# Steroid responsive cavernous sinus syndrome due to Rosai-Dorfman disease: beyond Tolosa-Hunt syndrome – a case report

**DOI:** 10.1186/s12883-021-02255-z

**Published:** 2021-07-05

**Authors:** Paulo Ribeiro Nóbrega, Pedro Gustavo Barros Rodrigues, Isabelle de Sousa Pereira, Carolina de Figueiredo Santos, Gunter Gerson, José Arnaldo Motta de Arruda, José Wagner Leonel Tavares Júnior, Pablo Picasso de Araújo Coimbra, Pedro Braga-Neto

**Affiliations:** 1 Division of Neurology, Department of Clinical Medicine, Universidade Federal do Ceará, Rua Capitão Francisco Pedro, 1290, 60430-370 Fortaleza, Brazil; 2 Hospital Infantil Albert Sabin, Fortaleza, Brazil; 3 Department of Pathology, Universidade Federal do Ceará, Fortaleza, Brazil; 4 Division of Neurosurgery, Department of Surgery, Universidade Federal do Ceará, Fortaleza, Brazil; 5 Uniclinic Diagnóstico por Imagem - UDI Fortaleza, Fortaleza, Brazil; 6 Center of Health Sciences, Universidade Estadual do Ceará, Fortaleza, Brazil

**Keywords:** Rosai-Dorfman Disease, Histiocytosis, Tolosa-Hunt Syndrome, Cavernous Sinus, Neuroimmunology, Case report

## Abstract

**Background:**

The term “Tolosa-Hunt syndrome” (THS) has been used to refer to painful ophthalmoplegia associated with nonspecific inflammation of the cavernous sinus and many processes can result in a similar clinical picture, including infectious, inflammatory and neoplastic diseases. Rosai-Dorfman disease (RDD) is a lymphoproliferative disorder that rarely affects the central nervous system. We report a case of isolated CNS Rosai-Dorfman disease involving the cavernous sinus and presenting as “Tolosa-Hunt syndrome”.

**Case presentation:**

Our patient presented with horizontal diplopia due to impairment of cranial nerves III, IV and VI and a stabbing/throbbing headache predominantly in the left temporal and periorbitary regions. There was a nonspecific enlargement of the left cavernous sinus on MRI and the patient had a dramatic response to steroids. Biopsy of a frontal meningeal lesion was compatible with RDD.

**Conclusions:**

We highlight the importance of including Rosai-Dorfman disease as a differential diagnosis in cavernous sinus syndrome and demonstrate a satisfactory long-term response to steroid treatment in this disease.

**Supplementary Information:**

The online version contains supplementary material available at 10.1186/s12883-021-02255-z.

## Background

The eponym “Tolosa-Hunt syndrome” (THS) was first used in 1966 referring to painful ophthalmoplegia associated with nonspecific inflammation of the cavernous sinus [1] and it remains a diagnosis of exclusion. It is a rare condition that affects one in one million people worldwide and many pathological processes can result in a similar clinical picture, including infectious, inflammatory and neoplastic diseases [2]. Some of the diseases that mimic THS may present a rapid response to steroids, which led to some authors calling for a retirement of the diagnostic eponym [3]. as it could be often misleading and postpone a diagnosis of a potentially life-threatening condition.

Rosai-Dorfman disease (RDD) is a lymphoproliferative disorder of unknown etiology. It is characterized by nonmalignant overproduction and accumulation of histiocytes in the lymph nodes and extra-nodal tissue. This disease has an incidence of approximately 100 new cases per year in the United States [4–9] and affects the central nervous system (CNS) in less than 5 % of cases. Most of these cases have isolated CNS involvement (> 80 %) and over 90 % involve the intracranial compartment [4, 10, 11], usually presenting as meningeal lesions that resemble meningiomas [11, 12]. We report a case of isolated CNS Rosai-Dorfman disease involving the cavernous sinus and presenting as “Tolosa-Hunt syndrome” with a dramatic response to steroids.

## Case presentation

A 38-year-old male patient presented with horizontal diplopia, divergent strabismus and ptosis of the left eyelid. He also had an intense stabbing/throbbing headache predominantly in the left temporal and periorbitary regions. Headache was continuous and demonstrated little response to common analgesics and nonsteroidal anti-inflammatories (NSAIDs). There was no complaint of nausea, vomiting, phonofobia or photophobia. The patient also complained of numbness in his left frontal and upper face (territory of ophthalmic and maxillary divisions of the trigeminal nerve).

There was a previous history of right-sided focal motor seizures, and a previous brain surgery 10 years ago for resection of a low grade tumour of the left frontal lobe (there was no pathological or immunohistochemistry report of that surgery available).

On examination the patient had complete third nerve palsy, sixth nerve palsy and loss of pinprick and thermic sensations in the territories of the Ophtalmic (V1) and Maxillary (V2) divisions of the trigeminal nerve on the left side. Other parts of the neurological examination, including cognition, motricity, reflexes and coordination were normal. There was no papilledema.

A clinical diagnosis of cavernous sinus syndrome was made, and the patient was started on prednisone 60 mg/day, with significant improvement of headache intensity, resolution of ptosis and improvement in adduction, elevation and partial improvement in abduction of the left eye. An additional movie file shows this in more detail [see Additional file 1].

**Fig. 1:**
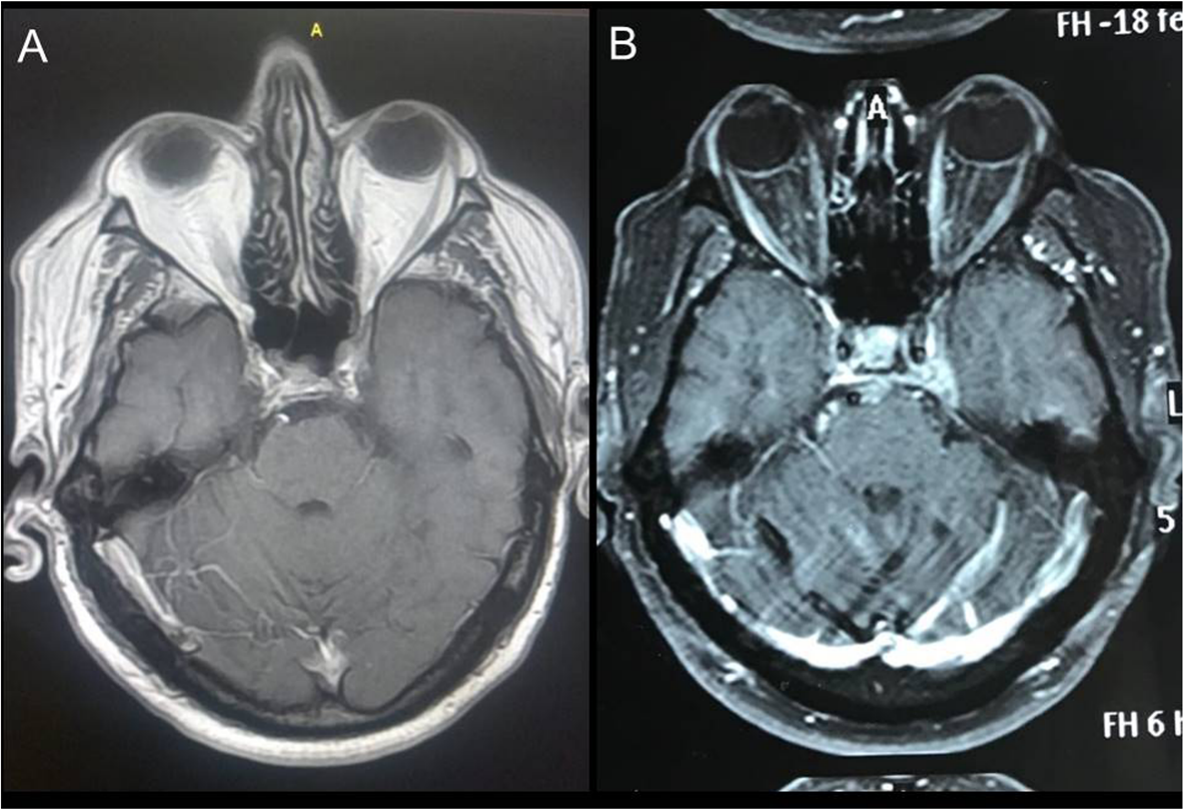
Neuroimaging findings in the patient with Rosai-Dorfman disease demonstrating: asymmetry of the cavernous sinuses in T1, with the left cavernous sinus larger than the right, and contrast enhancement of the left cavernous sinus

**Fig. 2:**
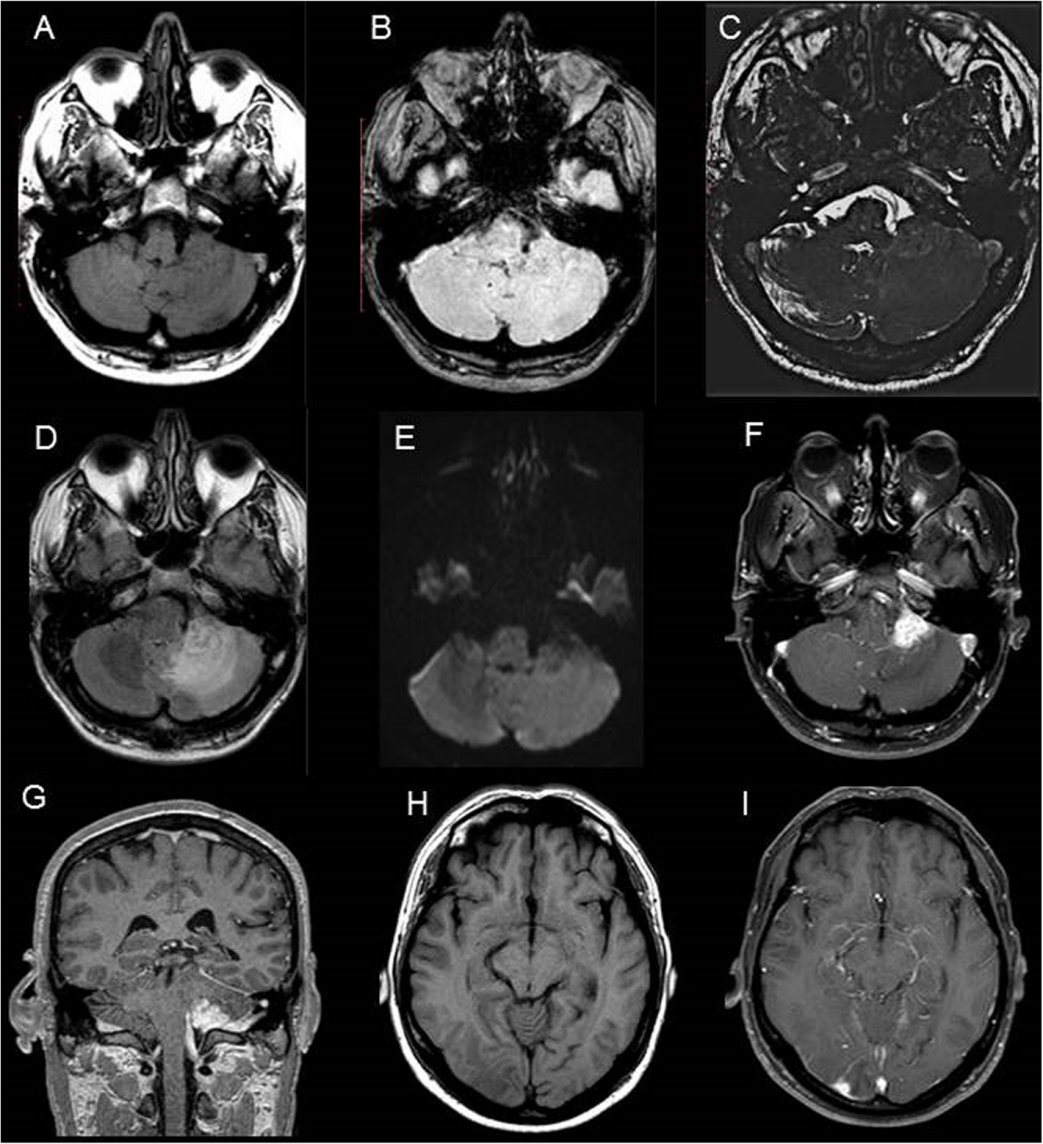
Neuroimaging findings in the patient with Rosai-Dorfman disease demonstrating extra-axial nodular image located in the left cerebellopontine angle cistern with isosignal in T1 and low signal in T2 / FLAIR with homogeneous contrast enhancement, surrounded by cerebellar edema, with dural tail and no restricted diffusion (**A**, **B** to **G**). In **H** and **I** there is a small extra-axial image with the same characteristics described above in the right temporal region

Brain magnetic resonance imaging (MRI) revealed an asymmetry of the cavernous sinuses, with the left cavernous sinus being larger than the right (Fig. 1). There were also four meningeal lesions with contrast enhancement and without calcification in the upper convexity adjacent to the left and right frontal lobes, in the left cerebellopontine angle and left posterior temporal region (Fig. 2).

**Fig. 3:**
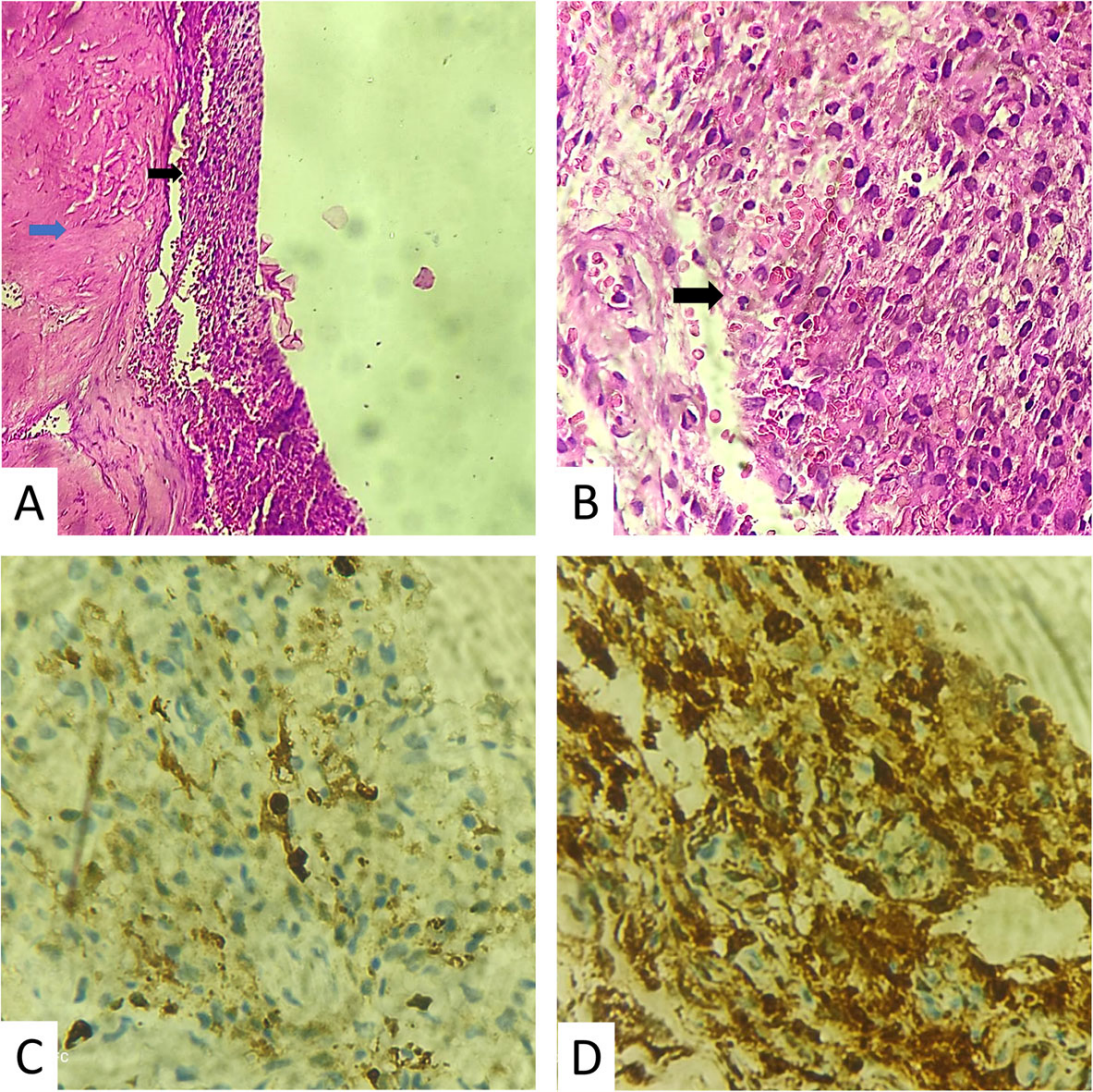
**A**- HE staining, 10x low magnification: Histological specimen show sections of fibrous and thickened dura mater (blue arrow) with extensive superficial infiltration by histiocytic cells (black arrow). **B** - HE staining, 20x intermediate magnification Histological sections show extensive superficial meningeal infiltration by histiocytes ( arrow ); absence of cell atypias, malignant cells or meningothelial neoplasia. **C** - Immunohistochemical reaction for CD68: diffuse positivity in lesion cells, confirming histiocytic aspect. **D** - Immunohistochemical reaction for S100: focal positivity in lesion cells, confirming histiocytic aspect. Other reactions performed were negative: CD1a and ALK (not shown)

A biopsy of the meningeal lesion in the frontal convexity was performed, and resulted in a meningeal fragment with histiocytic proliferation (Fig. 3). Immunohystochemical profile was negative for CD1a and ALK-1 and was positive for S100, CD68 (KP1 clone) and EMA (E29 clone), compatible with Rosai-Dorfman disease. Whole body computerized tomography (CT) scans did not reveal any other masses or lymphadenomegalies, characterizing an isolated CNS Rosai-Dorfman disease.

**Fig. 4:**
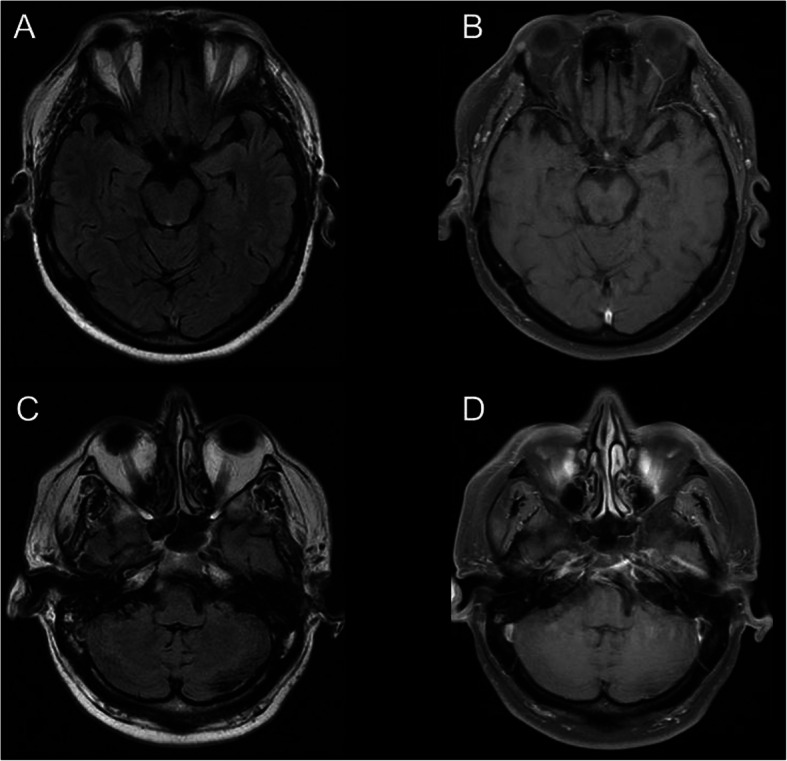
Neuroimaging findings in the patient with Rosai-Dorfman disease one year after the first examination demonstrating disappearance of the extra-axial nodular image located in the left cerebellopontine angle cistern and the right temporal region, as well as resolution of cavernous sinus asymmetry

The patient was treated with intravenous methylprednisolone 1 g/kg daily for five days, with significant improvement of the cavernous sinus syndrome. Maintenance treatment consisted of prednisone 10 mg/day after tapering. He is asymptomatic and has remained seizure-free for two years. Follow-up brain MRI has showed a decrease in size of meningeal lesions and of cavernous sinus asymmetry (Fig. 4).

## Discussion and conclusions

The term “Tolosa-Hunt syndrome” has been used to describe a syndrome of painful ophthalmoplegia characterized by periorbital or hemicranial pain associated with ipsilateral ocular motor nerve (third, fourth and sixth cranial nerves) palsies, oculosympathetic impairment, and trigeminal sensory loss in the distribution of the ophthalmic and/or maxillary divisions [2].

The disease is believed to be related to a non-specific inflammatory process in the region of the cavernous sinus and have a relapsing and remitting course with prompt response to systemic corticosteroid therapy [2]. There is an extensive and growing list of differential diagnoses, including IgG4-related disease [13], many of which may respond rapidly to steroids. Thus, the clinical picture, neuroimaging and even a dramatic response to steroids are not specific, and it remains a diagnosis of exclusion. Incorrect use of the eponym could therefore result in missing infective or neoplastic conditions and delaying appropriate treatment [3].

We described a patient which fulfilled the International Classification of Headache Disorders (ICHD) criteria for THS (Table 1) [14] and was found to have isolated CNS Rosai-Dorfmann disease.

**Table 1:** International Classification of Headache Disorders (ICHD-3) criteria for Tolosa-Hunt syndrome

DESCRIPTION
Unilateral orbital and periorbital pain associated with paresis of one or more of the third, fourth or sixth cranial nerves caused by a granulomatous inflammation in the cavernous sinus, superior orbital fissure or orbit.
**DIAGNOSTIC CRITERIA**
**A. Unilateral orbital or periorbital headache fulfilling criterion C.**
**B. Both of the following**: 1. Granulomatous inflammation of the cavernous sinus, superior orbital fissure or orbit, demonstrated by MR scan or biopsy. 2. Paresis of one or more of the ipsilateral third, fourth and/or sixth cranial nerves.
**C. Evidence of causation demonstrated by both of the following**: 1. Headache is ipsilateral to the granulomatous lesion. 2. Headache has preceded paresis of the third, fourth and/or sixth nerves by ≤2 weeks or developed with it.
**D. Not better accounted for by another ICHD-3 diagnosis.**
Comments: ► Some reported cases of 13.8 Tolosa-Hunt syndrome had additional involvement of the fifth nerve (commonly the first division) or optic, seventh or eighth nerves. Sympathetic innervation of the pupil is occasionally affected. ► Careful follow-up is required to exclude other causes of painful ophthalmoplegia such as tumours, vasculitis, basal meningitis, sarcoidosis or diabetes mellitus. ► Pain and paresis of 13.8 Tolosa-Hunt syndrome resolve when it is treated adequately with corticosteroids.

Rosai-Dorfman disease, or Sinus Histiocytosis with Massive Lymphadenopathy, was first described in 1969 by Rosai and Dorfman [8]. Its clinical presentation is very heterogenous and depends on the affected body sites, but it usually presents with bilateral, massive and painless cervical lymphadenopathy that might be associated with fever, weight loss, night sweats, polyclonal hypergammaglobulinemia, high erythrocyte sedimentation rate and leukocytosis [5–9, 15]. The most common extra-nodal sites are skin, orbit and eyelid, bone, central nervous system and upper respiratory tract [5–7, 11, 15–17]. Biopsy reveals accumulation of histiocytes positive for S-100 protein and for CD 68, and negative for CD1a on immunohistochemical examination [5–7]. Emperipolesis can occur, which consists in the presence of intact hematopoietic cells (mostly lymphocytes) inside histiocytes [5–7, 15]. This is a typical feature of systemic RDD, but may not always be present in isolated CNS disease [18].

Intracranial Rosai-Dorfman disease typically involves the meninges, presenting as an enhancing dural-based lesion with surrounding vasogenic edema mimicking a meningioma [19], which was also the case with our patient. It usually shows isointensity on T1-weighted MRI and isointensity with areas of hypointensity on T2-weighted or FLAIR imaging [19]. A dural tail is present in most cases, being absent in a single case report [20]. It has been suggested that the absence of internal calcification or haemorrhage on CT and the presence of areas of low intensity within the lesion on T2-weighted MRI can help in differentiating meningeal RDD from a meningioma [19, 20]. It may also eventually resemble lymphomas or metastases and biopsy is necessary for a histopathological diagnosis to be confirmed [17].

Symptoms of intracranial Rosai-Dorfman disease vary according to the site of the meninges affected. Headaches, seizures and focal neurological deficits due to mass effect and edema are the most common neurological symptoms, but cranial nerve dysfunction, cognitive decline and pituitary dysfunction may also occur [11].

In a recent review Li et al. found 12 previously published cases of RDD involving the cavernous sinus [10, 12, 29, 30, 21–28], but none of them reported fulfilling clinical criteria for THS, including a dramatic response to steroids [30]. This case report supports the inclusion of Rosai-Dorfman Disease in the extensive list of differential diagnoses for painful ophtalmoplegia syndrome.

The best strategy for management of primary CNS RDD is still uncertain, therefore treatment is aligned with the individualities of each case. Therapy can vary from surgery, corticosteroids, serolimus, radiotherapy, chemotherapy or immunomodulation. Surgery is considered the best option for unifocal disease or cases with symptomatic sinus, airway, cranial or spinal disease [9] and it has been suggested that radical resection should be the ideal surgical goal [15, 17, 31, 32]. In some cases, however, the lesions may invade or surround critical structures, such as the cavernous sinus in our case, the carotid artery, or adhere to the brain cortex, and may not be completely resectable. Response to steroids has been variable and often incomplete in previous reports [15, 32]. A recently published case of isolated meningeal RDD found complete remission with low-dose steroids [33]. The patient reported here had a dramatic response to steroids and remission was maintained after tapering with low-dose steroids for a prolonged time.

The definitive diagnosis in patients suspected to have “Tolosa-Hunt syndrome” is challenging as in many of these cases a potentially life-threatening disease may be the actual cause of the symptoms. We highlight in this case report the need to include Rosai-Dorfman disease as a differential diagnosis in cavernous sinus syndrome.

## Supplementary information

Additional file 1. Neurological examination in a patient with cavernous sinus syndrome due to Rosai-Dorfman disease after pulsed steroid therapy demonstrating left sixth nerve palsy.

## Data Availability

Not applicable.

## Abbreviations

CT: Computerized tomography.
CNS: Central nervous system.
ICHD: International Classification of Headache Disorders.
MRI: Magnetic resonance imaging.
NSAIDs: Nonsteroidal anti-inflammatories drugs.
RDD: Rosai-Dorfman disease.
THS: Tolosa-Hunt syndrome.
